# Psychotropics/Neuromodulators in the Treatment of Functional Gastrointestinal Disorders – Old Wine in New Bottles

**DOI:** 10.1192/j.eurpsy.2026.11037

**Published:** 2026-07-31

**Authors:** N. Rijavec

**Affiliations:** 1 University Psychiatric Clinic Ljubljana, University Psychiatric Clinic Ljubljana; 2 Department of Psychiatry, Faculty of Medicine, University of Ljubljana, Ljubljana, Slovenia

## Abstract

**Introduction:**

Functional gastrointestinal disorders (FGIDs), characterized by dysfunction in the bidirectional neurohumoral communication between the gastrointestinal tract and the central nervous system, are commonly comorbid with depression and anxiety disorders, which complicates the diagnosis, treatment, and outcomes of both conditions.

**Objectives:**

With this literature review, we aim to highlight the effectiveness of psychotropic medications, also known as neuromodulators, in treating FGIDs, both with and without the presence of mental disorders.

**Methods:**

A systematic literature review was conducted to examine the effects of psychotropic drugs (neuromodulators) on FGIDs. Searches were performed in PubMed using relevant keywords. Only studies focusing on the use of these treatments in relation to FGIDs were included.

**Results:**

Many psychotropic drugs (antidepressants, antipsychotics, and antiepileptics) are successfully used to treat FGIDs. In addition to their effects on the central nervous system (central processing of visceral pain stimuli and responses to them), they also exert peripheral effects by influencing the sensitivity and motility of the gastrointestinal organs as well as the tone of the sphincters. The term “neuromodulator” is used instead of “psychotropic” to reduce stigma, emphasize clinical effectiveness, and improve therapeutic adherence.

**Conclusions:**

Diagnosis and treatment of FGIDs is performed by gastroenterologists, while the psychiatrist acts as a consultant who diagnoses and treats psychiatric comorbidities and implements pharmacotherapy and psychotherapy. The following psychiatric approaches are recommended: when administering medication, the desired therapeutic effects of the drug should be utilized. Additionally, some side effects (e.g., sedation) can be beneficial. The rule is to “go slow and low”: monitor the clinical response and any bothersome side effects, adjust the dosage, switch medications, or augment treatment with a drug from a different class as needed. Psychotherapy should be applied when indicated. Treatment should be continued for an adequate duration, with emphasis on establishing a strong therapeutic relationship, educating the patient, and providing ongoing support.

**Disclosure of Interest:**

None Declared

